# Supplementary material for: Effect of goal-oriented prenatal education on birth preparedness, complication readiness and institutional delivery among semi-urban pregnant women in Nigeria: A quasi-experimental study
Source: PLoS One. 2023 Jul 31;18(7):e0289414. doi: 10.1371/journal.pone.0289414 (PMC10389740; doi:10.1371/journal.pone.0289414)
Supplement: Attachment — Submitted filename: Response to Reviewers.docx [file pone.0289414.s001.docx]

**Response to Reviewer’s Comments**

- The title page was added to the manuscript
- The second sentence of the introduction which was previously stated as “the maternal mortality ratio of lifetime risk has been reported to be as high as 462 per 100,000 live birth was restated to read “the lifetime risk of death from pregnancy and delivery-related complications have been reported to be as high as 462 per 100,000 live births”
- On line 68, the word “prevention” was replaced with “reduction”
- Line 69, a new reference was added. The same reference was also added to the list of references on lines 432 – 434.
- Line 93, a new reference was added. The same was added to the reference list on lines 453 – 455.
- Study design: The women were not randomly allocated to the intervention and the control group. The randomization was done at the level of local government. The two selected local governments were randomized into intervention and control groups – line 117. Therefore, since the randomization of the women into intervention and control groups was not done, it is a quasi-experimental study.
- Sampling technique: The sampling technique in the abstract section was revised as “Two Local Government Areas (LGAs) were randomly selected from the six semi-urban LGAs in Ibadan. These LGAs were randomized into an intervention and control group. Two Primary Healthcare Centres (PHCs) were randomly selected from each LGA, and 400 pregnant women who registered for antenatal care in the selected PHCs, and met the inclusion criteria were purposively selected to participate in the study” (lines 38 – 42).
- Client recruitment and data collection: The research assistants who did the recruitment also conducted the data collection. So, the data collectors were aware of which intervention group the women belong.
- Results: Table 1 has been revised based, and it has also been merged with table 2. However, tables 3,4, and 5 could not be merged because each of them is targeting different specific objectives of the study.
- A brief introductory paragraph was added as the first paragraph in the discussion section.
- The discussion section was revised to remove unnecessary repetition of results in the section.
- The first two statements under the limitation for the study was deleted as suggested.
- A section stating the strengths of the study was added (lines 396 – 402)
- The conclusion section was revised to be more focused and concise.
