# Supplementary material for: Effect of goal-oriented prenatal education on birth preparedness, complication readiness and institutional delivery among semi-urban pregnant women in Nigeria: A quasi-experimental study
Source: PLoS One. 2023 Jul 31;18(7):e0289414. doi: 10.1371/journal.pone.0289414 (PMC10389740; doi:10.1371/journal.pone.0289414)
Supplement: Attachment — Submitted filename: PONE-D-23-02382_R1_reviewer.docx [file pone.0289414.s002.docx]

Reviewer: Richard Kalisa Reviewer’s report:

Thank you for the opportunity to review this manuscript. This is well done and your research results are very encouraging if this type of approaches are applied in the country it is very likely to have an impact on reducing MMR.

Professional English editing is needed.

The authors should revise the introduction to clearly highlight the problem statement. Together, I would suggest the authors during their write up to consider using recent BPCR references from countries with similar maternal mortality rates.

The entire method section requires revision to improve readability by ensuring there is clear flow of information. Please include a discussion on inclusion and exclusion criteria. How many participants were not included due to not having a cell phone? Was this due to socio-economic status and was this an exclusion that could inherently bias the sample to wealthier and possibly more educated patients? Was the sample stratified by the number of previous pregnancies? This too could potentially bias the analysis. Line 127-133, the authors state: "adopted a validated, semi-structured/structured questionnaire from 'Monitoring BPCR tools and indicators for maternal and new-born health' developed by Johns Hopkins Program for International Education in Gynaecology and Obstetrics” They should indicate what adaptations or changes were made to the BP/CR tool in the local context.

The study does not give clear implications in the conclusion or recommendations. If the study provides similar findings as other studies, where is the problem? What is the explanation for this? What should be done about this situation? What should be the next logical step to address this issue?

The manuscript needs revision for some typographical errors as well need to revise the title

The authors should make sure you use [reference style](https://endnote.com/style_download/jama-journal-of-the-american-medical-association-ama-10th-edition/) for this journal
