# Supplementary material for: Effect of goal-oriented prenatal education on birth preparedness, complication readiness and institutional delivery among semi-urban pregnant women in Nigeria: A quasi-experimental study
Source: PLoS One. 2023 Jul 31;18(7):e0289414. doi: 10.1371/journal.pone.0289414 (PMC10389740; doi:10.1371/journal.pone.0289414)
Supplement: Attachment — Submitted filename: Response to reviewer_3.docx [file pone.0289414.s003.docx]

Response to reviewer’s comments

| **Reviewer’s Comments** | **Author’s Response** |
| --- | --- |
| Professional English editing is needed | Professional English editing was done |
| The authors should revise the introduction to clearly highlight the problem statement | The introduction section was reviewed to highlight problem statements |
| The method section requires revision to improve readability by ensuring there is clear flow of information | The method section was reviewed. |
| Include a discussion on inclusion and exclusion criteria | The inclusion criteria were discussed at the methods section |
| How many participants were not included due to not having a cell phone? Was this due to socio-economic status and was this an exclusion that could inherently bias the sample to wealthier and possibly more educated patients? | Using of a cell phone was not a criterium for inclusion. However, those that had cell phones had their phone number documented, while those who did not have had their husbands phone number documented to remind them of the intervention date and/or follow-up interview date (Line 187) as well as to monitor their date and site of delivery (Lines 210 – 214). |
| Was the sample stratified by the number of previous pregnancies? | The sample was not stratified by the number of previous pregnancies. |
| The authors state: "adopted a validated, semi-structured/structured questionnaire from 'Monitoring BPCR tools and indicators for maternal and new-born health' developed by Johns Hopkins Program for International Education in Gynaecology and Obstetrics” They should indicate what adaptations or changes were made to the BP/CR tool in the local context. | The validated questionnaire developed by Johns Hopkins Program for International Education in Gynaecology and Obstetrics was “adopted” and not “adapted” with little or no changes. Except for change of terminologies to simple terms that can be easily understood by the study participants |
| If the study provides similar findings as other studies | Comparison with similar previous studies were highlighted in the paper, with relevant implications. |
| The manuscript needs revision for some typographical errors | The manuscript was revised for some typographical errors |
| The authors should make sure you use [reference style](https://endnote.com/style_download/jama-journal-of-the-american-medical-association-ama-10th-edition/) for this journal | Reference style for the journal was ensured. |
| In the Methods section please include the informed consent statement to reflect whether "written or verbal" informed consent was obtained from all participants for inclusion in the study | “verbal” inform consent was obtained (Line 228) |
